# Supplementary material for: The molecular origin and taxonomy of mucinous ovarian carcinoma
Source: Nat Commun. 2019 Sep 2;10:3935. doi: 10.1038/s41467-019-11862-x (PMC6718426; doi:10.1038/s41467-019-11862-x)
Supplement: Supplementary file 1 — Supplementary Information [file 41467_2019_11862_MOESM1_ESM.pdf]

Supplementary Information for Cheasley *et al*.

The Molecular Origin and Taxonomy of Mucinous Ovarian Carcinoma.

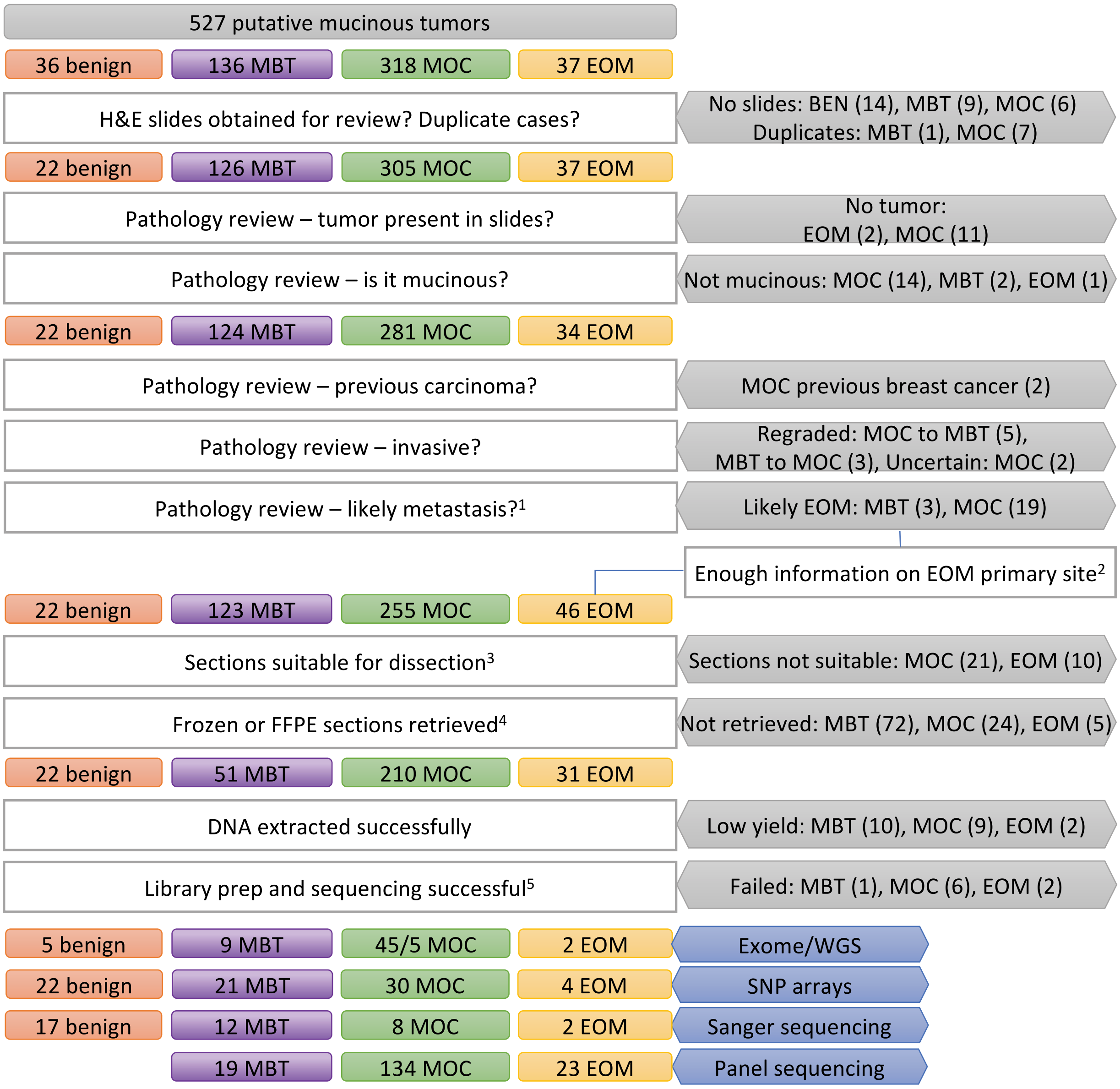

Supplementary Figure 1.
REMARK diagram showing selection for primary mucinous ovarian tumours for discovery and validation sequencing. MBT – mucinous borderline tumor; MOC – mucinous ovarian carcinoma; EOM – extra-ovarian metastatic disease

1. Metastatic disease was determined on an integrated assessment combining histopathological features, immunohistochemistry and clinical information including outcome. Cases where the metastatic status was uncertain were excluded as MOC.
2. Some cases were re-classed as EOM when the primary site could be determined.
3. Unsuitable sections included those with no invasive disease present (for MOC), scanty tumor unlikely to yield enough DNA, or a dissection unlikely to yield >50% tumor epithelial cells (e.g. highly infiltrative EOM cases).
4. Frozen tissues were prioritized, and DNA extracted from these was used for exome/whole genome (WGS) sequencing. Formalin-fixed, paraffin-embedded (FFPE) tissues were only requested from AOCS, COEUR, Queensland, Southern, VCB and Edinburgh as these were the largest collections and with the most detailed clinical information available. MBT and EOM cases were not prioritized for FFPE tissue collection.
5. Benign data as well as Sanger sequencing and SNP array data were pre-existing from our previous studies (Hunter et al., 2013, Ryland et al., 2015). Some MBT (n=8), EOM (n=2) and MOC (n=11) data were previously obtained by exome sequencing (Ryland et al., 2015). New EOM and MBT cases were prioritized for panel sequencing.

Source data for this figure provided in the Source Data file.

Supplementary Note 1

a. Survival analysis – source data in Supplementary Data 1

1. Univariate analysis of clinicopathological parameters across MBT, MOC and EOM

a. Supplementary Table 1 - Disease-specific overall survival

**Factor**		**HR**	**95% CI**	**P value**
Classification	MBT	1		
	MOC	6.24	1.93-20.15	0.002
	EOM	47.44	13.74-163.76	1.02E-09
Grade	MOC G1	1		
	MOC G2	1.17	0.58-2.34	0.66
	MOC G3	4.05	1.85-8.83	0.0004
	MBT	0.2	0.06-0.7	0.012
	EOM	10.3	4.94-21.47	4.99E-10
FIGO Stage	MBT	1		
	MOC I	3.27	0.96-11.11	0.057
	MOC II	4.88	0.98-24.35	0.053
	MOC III-IV	33.15	9.8-112.21	1.82E-08
	EOM	48.96	14.18-169.01	7.48E-10
Age (years)		1.01	0.99-1.03	0.158

Supplementary Figure 2 - Disease-specific overall survival

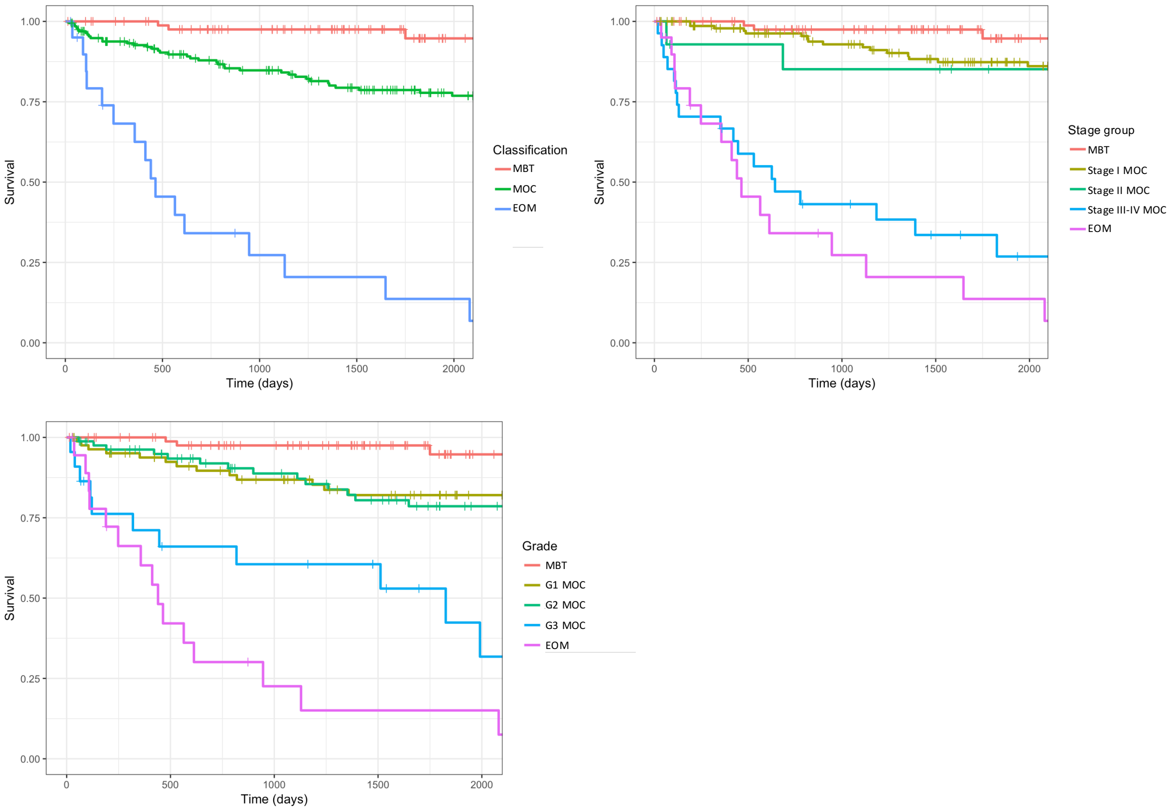

b. **Supplementary Table 2 - Progression-free survival**

**Factor**		**HR**	**95% CI**	**P value**
Classification	MBT	1		
	MOC	4.64	1.83-11.76	0.001
	EOM	34.5	12.46-95.49	9.33E-12
Grade	MOC G1	1		
	MOC G2	0.95	0.46-1.98	0.898
	MOC G3	4.22	1.98-9.02	0
	MBT	0.27	0.1-0.76	0.013
	EOM	10.6	4.99-22.52	8.41E-10
FIGO Stage	MBT	1		
	MOC I	2.59	0.97-6.94	0.058
	MOC II	5.01	1.34-18.71	0.017
	MOC III-IV	24.01	8.76-65.78	6.37E-10
	EOM	35.88	12.93-99.54	6.15E-12
Age (years)		0.99	0.97-1.01	0.447

Supplementary Figure 3 - Progression-free survival

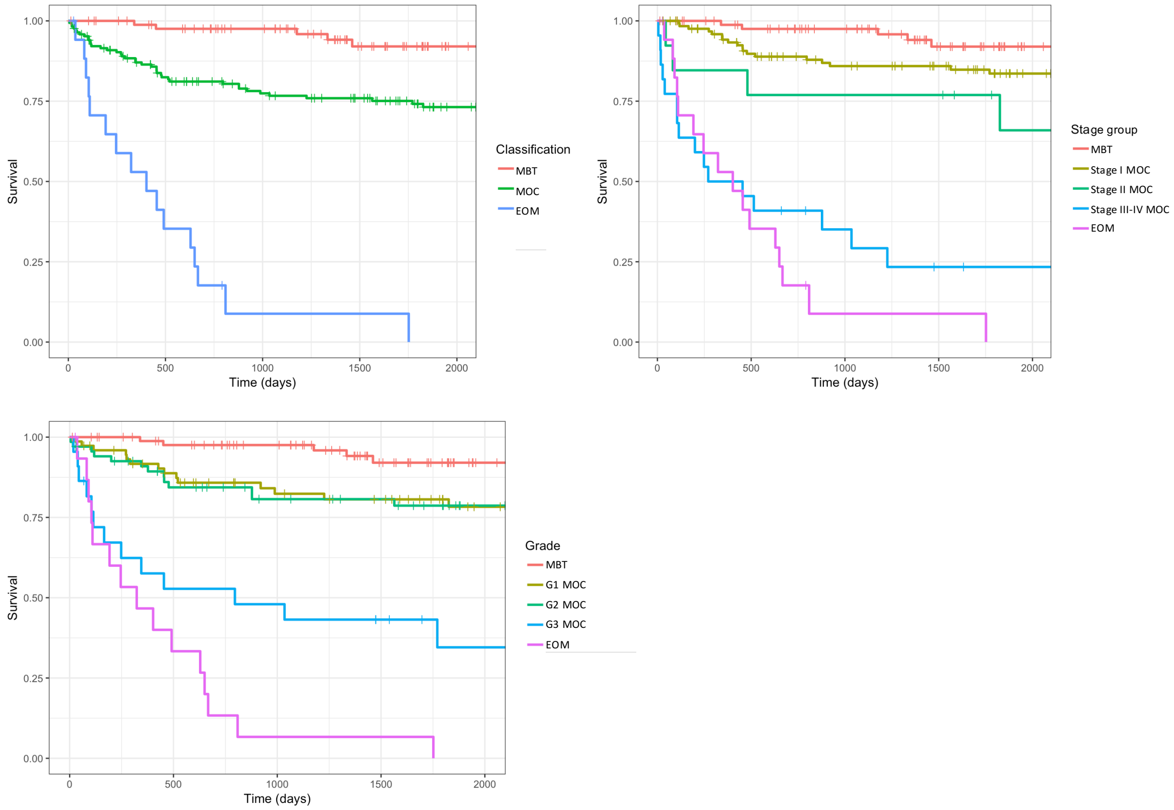

2. Univariate analysis of clinicopathological and genetic parameters within MOC

a. **Supplementary Table 3 - Disease-specific overall survival, MOC only**

**Factor**		**HR**	**95% CI**	**P value**
Grade	MOC G1	1		
	MOC G2	1.11	0.55-2.25	0.772
	MOC G3	3.92	1.79-8.55	0.001
FIGO Stage	I	1		
	II	1.48	0.44-5.02	0.526
	III	9.8	5.01-19.15	2.49E-11
	IV	12.03	2.77-52.3	0.001
Age (years)	Age	1.01	0.99-1.03	0.299
CN type	Complex	1		
	Complex (whole chr)	0.2	0.03-1.53	0.122
	Complex + amps	0.65	0.34-1.26	0.2
	Simple	0.12	0.03-0.51	0.004
	Simple + amp	0.24	0.05-1.03	0.055
FGA	≤10%	1		
	>10%	5.14	1.83-14.42	0.002
KRAS	MUT	1		
	WT	0.53	0.26-1.12	0.097
TP53	MUT	1		
	WT	0.89	0.46-1.73	0.737
ERBB2 amplification	Amplified	1		
	Not amplified	2.14	0.95-4.82	0.066
CDKN2A Loss/LOH	No	1		
	Yes	2.88	1.21-6.86	0.017
chr9p	Not amplified	1		
	Amplified	2.2487	1.04-4.863	0.040

Supplementary Figure 4 - Disease-specific overall survival, MOC only

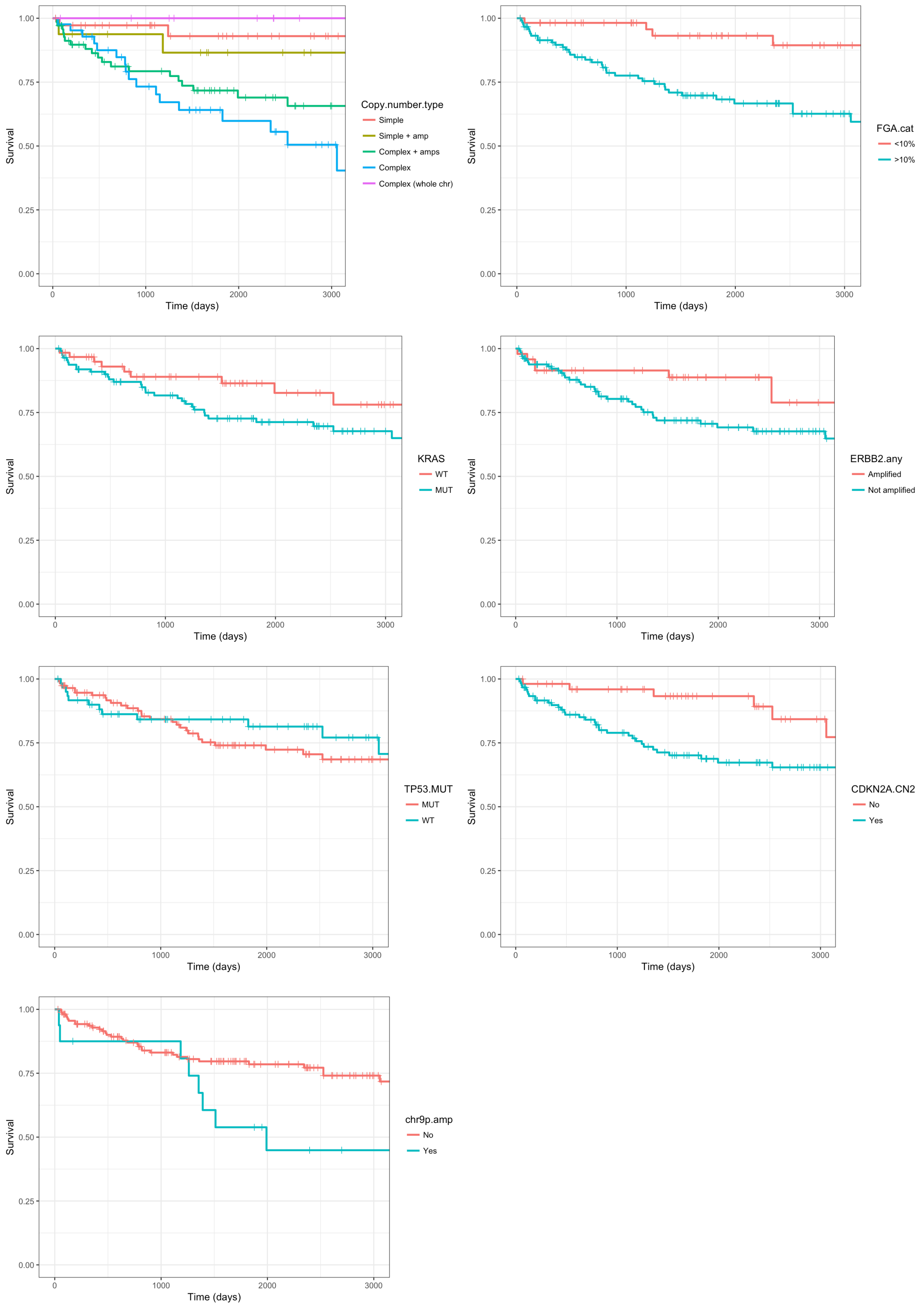

b. Supplementary Table 4 - Progression-free survival, MOC only

Factor		HR	95% CI	P value
Grade	MOC G1	1		
	MOC G2	0.91	0.43-1.9	0.792
	MOC G3	4.1	1.92-8.78	0.0003
FIGO Stage	I	1		
	II	1.96	0.67-5.75	0.218
	III	8.43	4.19-16.94	2.19E-09
	IV	10.31	2.38-44.72	0.002
Age (years)	Age	0.99	0.97-1.01	0.306
CN type	Complex	1		
	Complex (whole chr)	0.25	0.03-1.91	0.184
	Complex + amps	0.79	0.4-1.56	0.499
	Simple	0.08	0.01-0.59	0.013
	Simple + amp	0.55	0.18-1.66	0.291
FGA	≤10%	1		
	>10%	4.83	1.72-13.6	0.003
KRAS	MUT	1		
	WT	0.53	0.25-1.11	0.091
TP53	MUT	1		
	WT	0.72	0.35-1.47	0.368
ERBB2 amplification	Amplified	1		
	Not amplified	1.73	0.8-3.75	0.162
CDKN2A Loss/LOH	No	1		
	Yes	3.44	1.35-8.78	0.010
chr9p	Not amplified	1		
	Amplified	2.39	1.10-5.17	0.027

Supplementary Figure 5 - Progression-free survival, MOC only

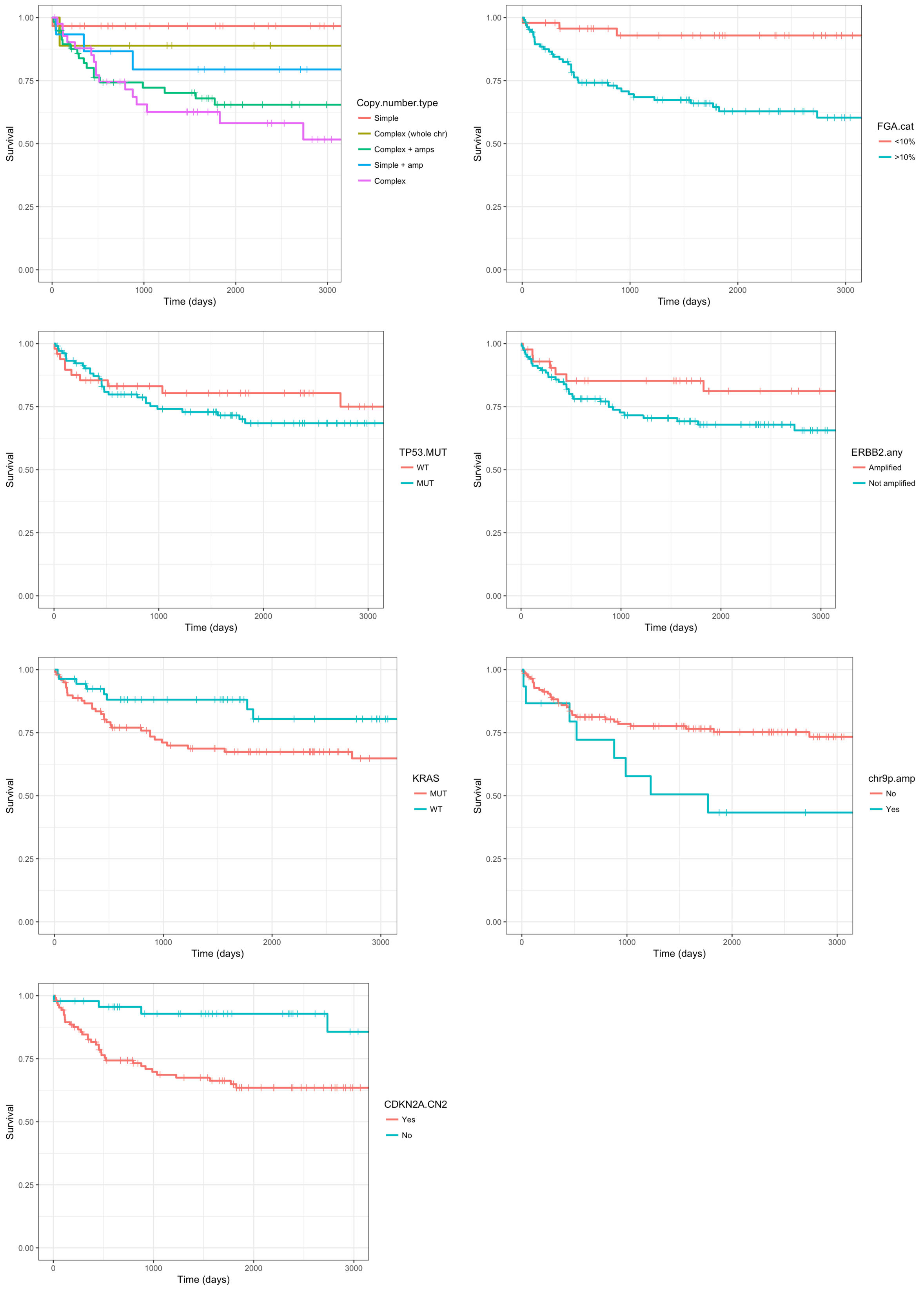

3. Akaike information criterion (AIC) multivariate modelling within MOC for disease-specific overall survival

```
Scope=list(upper=~(chr9p.amp+Copy.number.type+FGA.cat+CDKN2A.CN2+Grade+Stag
e.group+ERBB2.any+KRAS+TP53.MUT)^2,lower~1)
phm_0 = coxph(Surv(OS.days,DSS.Status)~1,na.action=na.exclude)
phm_f = stepAIC(phm_0,Scope,direction="both")

## Start:  AIC=343.38
## Surv(OS.days, DSS.Status) ~ 1
##
##                     Df    AIC
## + Stage.group        3 319.56
## + FGA.cat            1 333.77
## + Copy.number.type   4 337.21
## + Grade              2 337.73
## + CDKN2A.CN2         1 339.64
## + chr9p.amp          1 341.57
## + KRAS               1 342.27
## + ERBB2.any          1 342.33
## <none>                 343.38
## + TP53.MUT           1 345.11
##
## Step:  AIC=319.56
## Surv(OS.days, DSS.Status) ~ Stage.group
##
##                     Df    AIC
## + CDKN2A.CN2         1 311.64
## + Copy.number.type   4 312.53
## + FGA.cat            1 312.94
## + KRAS               1 317.98
## + TP53.MUT           1 318.59
## + chr9p.amp          1 318.65
## + ERBB2.any          1 319.50
## <none>                 319.56
## + Grade              2 321.13
## - Stage.group        3 343.38
##
## Step:  AIC=311.64
## Surv(OS.days, DSS.Status) ~ Stage.group + CDKN2A.CN2
##
##                           Df    AIC
## + FGA.cat                  1 308.45
## + Copy.number.type         4 309.89
## + ERBB2.any                1 310.15
## + KRAS                     1 310.19
## + Grade                    2 310.79
## <none>                       311.64
## + chr9p.amp                1 313.09
## + TP53.MUT                 1 313.42
## + CDKN2A.CN2:Stage.group   2 314.67
## - CDKN2A.CN2               1 319.56
## - Stage.group              3 339.64
##
## Step:  AIC=308.45
## Surv(OS.days, DSS.Status) ~ Stage.group + CDKN2A.CN2 + FGA.cat
##
##                           Df    AIC
## + ERBB2.any                1 305.35
## + KRAS                     1 305.85
```

```
## + Grade                      2 307.05
## <none>                         308.45
## + chr9p.amp                  1 309.64
## + FGA.cat:CDKN2A.CN2         1 309.74
## + TP53.MUT                   1 310.40
## + Copy.number.type           4 311.01
## + FGA.cat:Stage.group        2 311.47
## + CDKN2A.CN2:Stage.group     2 311.47
## - FGA.cat                    1 311.64
## - CDKN2A.CN2                 1 312.94
## - Stage.group                3 332.72
##
## Step:  AIC=305.35
## Surv(OS.days, DSS.Status) ~ Stage.group + CDKN2A.CN2 + FGA.cat +
##     ERBB2.any
##
##                             Df    AIC
## + Grade                      2 304.28
## + KRAS                       1 304.73
## <none>                         305.35
## + FGA.cat:CDKN2A.CN2         1 306.56
## + chr9p.amp                  1 306.77
## + FGA.cat:ERBB2.any          1 306.80
## + CDKN2A.CN2:ERBB2.any       1 307.15
## + TP53.MUT                   1 307.30
## + Copy.number.type           4 308.42
## + CDKN2A.CN2:Stage.group     2 308.45
## - ERBB2.any                  1 308.45
## + FGA.cat:Stage.group        2 308.50
## + Stage.group:ERBB2.any      2 308.76
## - FGA.cat                    1 310.15
## - CDKN2A.CN2                 1 311.02
## - Stage.group                3 328.81
##
## Step:  AIC=304.28
## Surv(OS.days, DSS.Status) ~ Stage.group + CDKN2A.CN2 + FGA.cat +
##     ERBB2.any + Grade
##
##                             Df    AIC
## + CDKN2A.CN2:Grade           2 296.98
## + KRAS                       1 302.50
## + Grade:Stage.group          4 303.86
## <none>                         304.28
## + FGA.cat:CDKN2A.CN2         1 305.32
## - Grade                      2 305.35
## + Copy.number.type           4 305.55
## + chr9p.amp                  1 305.65
## + FGA.cat:ERBB2.any          1 305.73
## + CDKN2A.CN2:ERBB2.any       1 306.02
## + TP53.MUT                   1 306.05
## + FGA.cat:Grade              2 306.24
## + CDKN2A.CN2:Stage.group     2 306.78
## - ERBB2.any                  1 307.05
## + Grade:ERBB2.any            2 307.22
## + Stage.group:ERBB2.any      2 307.69
## + FGA.cat:Stage.group        2 307.77
## - FGA.cat                    1 309.68
## - CDKN2A.CN2                 1 312.28
## - Stage.group                3 325.73
##
## Step:  AIC=296.98
```

```
## Surv(OS.days, DSS.Status) ~ Stage.group + CDKN2A.CN2 + FGA.cat +
##     ERBB2.any + Grade + CDKN2A.CN2:Grade
##
##                           Df    AIC
## <none>                       296.98
## + Grade:Stage.group        4 297.07
## + FGA.cat:Grade            1 297.88
## + KRAS                     1 298.18
## + FGA.cat:ERBB2.any        1 298.51
## + TP53.MUT                 1 298.53
## + chr9p.amp                1 298.63
## + CDKN2A.CN2:ERBB2.any     1 298.87
## + FGA.cat:CDKN2A.CN2       1 298.96
## + Stage.group:ERBB2.any    2 300.10
## + Grade:ERBB2.any          2 300.41
## + FGA.cat:Stage.group      2 300.52
## + CDKN2A.CN2:Stage.group   2 300.82
## - ERBB2.any                1 302.29
## + Copy.number.type         4 302.36
## - FGA.cat                  1 302.97
## - CDKN2A.CN2:Grade         2 304.28
## - Stage.group              3 320.93
```

```
summary(phm_f)
coxph(formula = Surv(OS.days, DSS.Status) ~ Stage.group + CDKN2A.CN2 +
## coxph(formula = Surv(OS.days, DSS.Status) ~ Stage.group + CDKN2A.CN2 +
##     FGA.cat + ERBB2.any + Grade + CDKN2A.CN2:Grade, na.action =
na.exclude)
##
##   n= 159, number of events= 37
##
##                              coef  exp(coef)   se(coef)      z  Pr(>|z|)
## Stage.group2           -6.169e-01  5.396e-01  8.304e-01 -0.743   0.4576
## Stage.group3            2.250e+00  9.486e+00  4.000e-01  5.625 1.85e-08
## Stage.group4            5.062e-01  1.659e+00  8.355e-01  0.606   0.5446
## CDKN2A.CN2Yes           2.235e-01  1.250e+00  5.906e-01  0.378   0.7051
## FGA.cat>10%             1.491e+00  4.441e+00  5.861e-01  2.543   0.0110
## ERBB2.anyNot amplified  1.147e+00  3.149e+00  4.679e-01  2.451   0.0142
## Grade2                 -1.747e+00  1.742e-01  1.118e+00 -1.562   0.1182
## Grade3                 -1.896e+01  5.819e-09  5.717e+03 -0.003   0.9974
## CDKN2A.CN2Yes:Grade2    1.411e+00  4.099e+00  1.201e+00  1.174   0.2402
## CDKN2A.CN2Yes:Grade3    2.018e+01  5.809e+08  5.717e+03  0.004   0.9972
##
## Stage.group2
## Stage.group3           ***
## Stage.group4
## CDKN2A.CN2Yes
## FGA.cat>10%            *
## ERBB2.anyNot amplified *
## Grade2
## Grade3
## CDKN2A.CN2Yes:Grade2
## CDKN2A.CN2Yes:Grade3
## ---
## Signif. codes:  0 '***' 0.001 '**' 0.01 '*' 0.05 '.' 0.1 ' ' 1
##
##                        exp(coef) exp(-coef) lower .95 upper .95
## Stage.group2           5.396e-01  1.853e+00   0.10599     2.748
## Stage.group3           9.486e+00  1.054e-01   4.33160    20.775
## Stage.group4           1.659e+00  6.028e-01   0.32261     8.531
```

```
## CDKN2A.CN2Yes          1.250e+00  7.997e-01   0.39295     3.979
## FGA.cat>10%            4.441e+00  2.252e-01   1.40781    14.008
## ERBB2.anyNot amplified 3.149e+00  3.176e-01   1.25850     7.877
## Grade2                 1.742e-01  5.739e+00   0.01946     1.560
## Grade3                 5.819e-09  1.719e+08   0.00000       Inf
## CDKN2A.CN2Yes:Grade2   4.099e+00  2.439e-01   0.38925    43.174
## CDKN2A.CN2Yes:Grade3   5.809e+08  1.722e-09   0.00000       Inf
##
## Concordance= 0.84  (se = 0.05 )
## Rsquare= 0.341   (max possible= 0.885 )
## Likelihood ratio test= 66.41  on 10 df,   p=2.18e-10
## Wald test            = 59.79  on 10 df,   p=3.969e-09
## Score (logrank) test = 91.94  on 10 df,   p=2.22e-15
```

4. Akaike information criterion (AIC) multivariate modelling within MOC for progression-free survival

```
Scope=list(upper=~(chr9p.amp+Copy.number.type+FGA.cat+CDKN2A.CN2+Grade+Stag
e.group+ERBB2.any+KRAS+TP53.MUT)^2,lower~1)
phm_0 = coxph(Surv(PFS.days,PFS.Status)~1,na.action=na.exclude)
phm_f = stepAIC(phm_0,Scope,direction="both")
```

```
## Start:  AIC=334.04
## Surv(PFS.days, PFS.Status) ~ 1
##
##                    Df    AIC
## + Stage.group       3 317.21
## + Grade             2 322.66
## + FGA.cat           1 325.64
## + Copy.number.type  4 329.09
## + CDKN2A.CN2        1 329.12
## + chr9p.amp         1 332.17
## + KRAS              1 333.01
## <none>                334.04
## + TP53.MUT          1 334.48
## + ERBB2.any         1 334.68
##
## Step:  AIC=317.21
## Surv(PFS.days, PFS.Status) ~ Stage.group
##
##                    Df    AIC
## + CDKN2A.CN2        1 310.71
## + FGA.cat           1 311.71
## + Copy.number.type  4 313.32
## + Grade             2 315.08
## + TP53.MUT          1 315.56
## + KRAS              1 315.79
## + chr9p.amp         1 316.71
## <none>                317.21
## + ERBB2.any         1 318.80
## - Stage.group       3 334.04
##
## Step:  AIC=310.71
## Surv(PFS.days, PFS.Status) ~ Stage.group + CDKN2A.CN2
##
##                          Df    AIC
## + Grade                   2 305.93
## + FGA.cat                 1 307.96
## <none>                      310.71
## + KRAS                    1 310.79
```

```
## + ERBB2.any               1 311.34
## + TP53.MUT                1 311.56
## + Copy.number.type        4 311.80
## + chr9p.amp               1 312.13
## + CDKN2A.CN2:Stage.group  2 313.46
## - CDKN2A.CN2              1 317.21
## - Stage.group             3 329.12
##
## Step:  AIC=305.93
## Surv(PFS.days, PFS.Status) ~ Stage.group + CDKN2A.CN2 + Grade
##
##                          Df    AIC
## + CDKN2A.CN2:Grade        2 300.45
## + FGA.cat                 1 302.39
## + Copy.number.type        4 305.10
## + TP53.MUT                1 305.32
## + KRAS                    1 305.48
## <none>                      305.93
## + ERBB2.any               1 306.73
## + Grade:Stage.group       4 307.06
## + chr9p.amp               1 307.40
## + CDKN2A.CN2:Stage.group  2 308.58
## - Grade                   2 310.71
## - CDKN2A.CN2              1 315.08
## - Stage.group             3 317.67
##
## Step:  AIC=300.45
## Surv(PFS.days, PFS.Status) ~ Stage.group + CDKN2A.CN2 + Grade +
##     CDKN2A.CN2:Grade
##
##                          Df    AIC
## + FGA.cat                 1 296.93
## <none>                      300.45
## + TP53.MUT                1 300.58
## + ERBB2.any               1 300.87
## + KRAS                    1 301.02
## + Grade:Stage.group       4 301.95
## + chr9p.amp               1 302.08
## + Copy.number.type        4 302.57
## + CDKN2A.CN2:Stage.group  2 304.06
## - CDKN2A.CN2:Grade        2 305.93
## - Stage.group             3 315.32
##
## Step:  AIC=296.93
## Surv(PFS.days, PFS.Status) ~ Stage.group + CDKN2A.CN2 + Grade +
##     FGA.cat + CDKN2A.CN2:Grade
##
##                          Df    AIC
## + ERBB2.any               1 296.88
## <none>                      296.93
## + KRAS                    1 297.31
## + Grade:Stage.group       4 297.44
## + chr9p.amp               1 298.43
## + TP53.MUT                1 298.67
## + Copy.number.type        4 298.72
## + FGA.cat:CDKN2A.CN2      1 298.78
## + FGA.cat:Stage.group     2 299.34
## + FGA.cat:Grade           2 299.76
## - FGA.cat                 1 300.45
## + CDKN2A.CN2:Stage.group  2 300.69
## - CDKN2A.CN2:Grade        2 302.39
```

```
## - Stage.group               3 311.27
##
## Step:  AIC=296.88
## Surv(PFS.days, PFS.Status) ~ Stage.group + CDKN2A.CN2 + Grade +
##     FGA.cat + ERBB2.any + CDKN2A.CN2:Grade
##
##                            Df    AIC
## + FGA.cat:ERBB2.any         1 294.77
## <none>                        296.88
## - ERBB2.any                 1 296.93
## + Grade:Stage.group         4 297.61
## + CDKN2A.CN2:ERBB2.any      1 297.69
## + TP53.MUT                  1 298.00
## + KRAS                      1 298.06
## + Copy.number.type          4 298.34
## + chr9p.amp                 1 298.46
## + FGA.cat:CDKN2A.CN2        1 298.60
## + FGA.cat:Stage.group       2 299.41
## + FGA.cat:Grade             2 299.62
## + Grade:ERBB2.any           2 300.27
## + Stage.group:ERBB2.any     2 300.37
## + CDKN2A.CN2:Stage.group    2 300.65
## - FGA.cat                   1 300.87
## - CDKN2A.CN2:Grade          2 302.58
## - Stage.group               3 310.27
##
## Step:  AIC=294.77
## Surv(PFS.days, PFS.Status) ~ Stage.group + CDKN2A.CN2 + Grade +
##     FGA.cat + ERBB2.any + CDKN2A.CN2:Grade + FGA.cat:ERBB2.any
##
##                            Df    AIC
## + FGA.cat:Stage.group       2 293.35
## + Grade:Stage.group         4 293.58
## <none>                        294.77
## + KRAS                      1 296.03
## + chr9p.amp                 1 296.21
## + TP53.MUT                  1 296.24
## + FGA.cat:CDKN2A.CN2        1 296.44
## + CDKN2A.CN2:ERBB2.any      1 296.75
## - FGA.cat:ERBB2.any         1 296.88
## + FGA.cat:Grade             2 297.58
## + Copy.number.type          4 298.06
## + CDKN2A.CN2:Stage.group    2 298.53
## + Stage.group:ERBB2.any     2 298.59
## + Grade:ERBB2.any           2 298.72
## - CDKN2A.CN2:Grade          2 300.07
## - Stage.group               3 308.79
##
## Step:  AIC=293.35
## Surv(PFS.days, PFS.Status) ~ Stage.group + CDKN2A.CN2 + Grade +
##     FGA.cat + ERBB2.any + CDKN2A.CN2:Grade + FGA.cat:ERBB2.any +
##     Stage.group:FGA.cat
##
##                            Df    AIC
## <none>                        293.35
## + FGA.cat:Grade             1 294.71
## - Stage.group:FGA.cat       2 294.77
## + KRAS                      1 294.80
## + FGA.cat:CDKN2A.CN2        1 295.16
## + TP53.MUT                  1 295.20
## + chr9p.amp                 1 295.21
```

```
## + Grade:Stage.group       4 295.33
## + CDKN2A.CN2:ERBB2.any    1 295.34
## + CDKN2A.CN2:Stage.group  2 297.12
## + Copy.number.type        4 297.17
## + Stage.group:ERBB2.any   2 297.24
## + Grade:ERBB2.any         2 297.29
## - FGA.cat:ERBB2.any       1 299.41
## - CDKN2A.CN2:Grade        2 299.58
```

```
summary(phm_f)
## Call:
## coxph(formula = Surv(PFS.days, PFS.Status) ~ Stage.group + CDKN2A.CN2 +
##     Grade + FGA.cat + ERBB2.any + CDKN2A.CN2:Grade + FGA.cat:ERBB2.any +
##     Stage.group:FGA.cat, na.action = na.exclude)
##
##   n= 143, number of events= 36
##    (16 observations deleted due to missingness)
##
##                                          coef  exp(coef)   se(coef)
z
## Stage.group2                        7.374e-01  2.090e+00  4.212e+04
0.000
## Stage.group3                        2.070e+01  9.776e+08  4.885e+03
0.004
## Stage.group4                        4.462e-01  1.562e+00  8.182e-01
0.545
## CDKN2A.CN2Yes                       1.741e-01  1.190e+00  6.248e-01
0.279
## Grade2                             -1.673e+00  1.876e-01  1.151e+00 -
1.454
## Grade3                             -1.991e+01  2.251e-09  1.001e+04 -
0.002
## FGA.cat>10%                        -4.541e-01  6.350e-01  8.886e-01 -
0.511
## ERBB2.anyNot amplified             -1.880e+01  6.850e-09  4.885e+03 -
0.004
## CDKN2A.CN2Yes:Grade2                1.214e+00  3.367e+00  1.224e+00
0.992
## CDKN2A.CN2Yes:Grade3                2.118e+01  1.577e+09  1.001e+04
0.002
## FGA.cat>10%:ERBB2.anyNot amplified  1.976e+01  3.805e+08  4.885e+03
0.004
## Stage.group2:FGA.cat>10%           -8.106e-01  4.446e-01  4.212e+04
0.000
## Stage.group3:FGA.cat>10%           -1.891e+01  6.129e-09  4.885e+03 -
0.004
## Stage.group4:FGA.cat>10%                   NA         NA  0.000e+00
NA
##                                    Pr(>|z|)
## Stage.group2                          1.000
## Stage.group3                          0.997
## Stage.group4                          0.585
## CDKN2A.CN2Yes                         0.781
## Grade2                                0.146
## Grade3                                0.998
## FGA.cat>10%                           0.609
## ERBB2.anyNot amplified                0.997
## CDKN2A.CN2Yes:Grade2                  0.321
## CDKN2A.CN2Yes:Grade3                  0.998
## FGA.cat>10%:ERBB2.anyNot amplified    0.997
## Stage.group2:FGA.cat>10%              1.000
```

```
## Stage.group3:FGA.cat>10%               0.997
## Stage.group4:FGA.cat>10%                  NA
##
##                                   exp(coef) exp(-coef) lower .95
## Stage.group2                      2.090e+00  4.784e-01   0.00000
## Stage.group3                      9.776e+08  1.023e-09   0.00000
## Stage.group4                      1.562e+00  6.400e-01   0.31433
## CDKN2A.CN2Yes                     1.190e+00  8.402e-01   0.34973
## Grade2                            1.876e-01  5.331e+00   0.01967
## Grade3                            2.251e-09  4.443e+08   0.00000
## FGA.cat>10%                       6.350e-01  1.575e+00   0.11129
## ERBB2.anyNot amplified            6.850e-09  1.460e+08   0.00000
## CDKN2A.CN2Yes:Grade2              3.367e+00  2.970e-01   0.30598
## CDKN2A.CN2Yes:Grade3              1.577e+09  6.343e-10   0.00000
## FGA.cat>10%:ERBB2.anyNot amplified 3.805e+08  2.628e-09   0.00000
## Stage.group2:FGA.cat>10%          4.446e-01  2.249e+00   0.00000
## Stage.group3:FGA.cat>10%          6.129e-09  1.632e+08   0.00000
## Stage.group4:FGA.cat>10%                 NA         NA        NA
##                                   upper .95
## Stage.group2                            Inf
## Stage.group3                            Inf
## Stage.group4                          7.766
## CDKN2A.CN2Yes                         4.050
## Grade2                                1.789
## Grade3                                  Inf
## FGA.cat>10%                           3.623
## ERBB2.anyNot amplified                  Inf
## CDKN2A.CN2Yes:Grade2                 37.060
## CDKN2A.CN2Yes:Grade3                    Inf
## FGA.cat>10%:ERBB2.anyNot amplified       Inf
## Stage.group2:FGA.cat>10%                Inf
## Stage.group3:FGA.cat>10%                Inf
## Stage.group4:FGA.cat>10%                 NA
##
## Concordance= 0.839  (se = 0.05 )
## Rsquare= 0.373   (max possible= 0.903 )
## Likelihood ratio test= 66.69  on 13 df,   p=3.241e-09
## Wald test            = 42.53  on 13 df,   p=5.369e-05
## Score (logrank) test = 88.13  on 13 df,   p=3.187e-13
```

b. Correlation of genetic events with grade and classification – source data in Supplementary Data 1

Supplementary Table 5 Correlation with Classification

		BEN	MBT	MOC	EOM	p value (FET*)
**KRAS**	MUT	12 (54.5%)	28 (71.8%)	124 (63.6%)	11 (40.7%)	0.063
	WT	10 (45.5%)	11 (28.2%)	71 (36.4%)	16 (59.3%)	
**BRAF**	MUT	0 (0.0%)	4 (10.3%)	18 (9.4%)	2 (7.7%)	0.563
	WT	22 (100.0%)	35 (89.7%)	174 (90.6%)	24 (92.3%)	
**TP53**	MUT	2 (9.1%)	7 (17.9%)	124 (63.6%)	11 (40.7%)	**<0.001**
	WT	20 (90.9%)	32 (82.1%)	71 (36.4%)	16 (59.3%)	
**CDKN2A inactivating**	Yes	5 (29.4%)	20 (51.3%)	103 (52.8%)	7 (25.9%)	0.016
	No	12 (70.6%)	19 (48.7%)	92 (47.2%)	20 (74.1%)	
**CDKN2A any (CN or**	Yes	12 (54.5%)	25 (64.1%)	151 (77.4%)	7 (25.9%)	**<0.001**
**mutation)**	No	10 (45.5%)	14 (35.9%)	44 (22.6%)	20 (74.1%)	
**CDKN2A Loss/LOH**	Loss	12 (54.5%)	22 (56.4%)	136 (69.7%)	5 (18.5%)	**<0.001**
	No Loss	10 (45.5%)	17 (43.6%)	59 (30.3%)	22 (81.5%)	
**ERBB2**	Amplified	0 (0.0%)	4 (3.7%)	52 (26.0%)	2 (6.5%)	**<0.001**
	Not amplified	22 (100.0%)	104 (96.3%)	148 (74.0%)	29 (93.5%)	
**Copy number type**	Complex	0 (0.0%)	5 (12.8%)	45 (23.1%)	7 (25.9%)	**<0.001^**
	Complex (whole chr)	0 (0.0%)	1 (2.6%)	10 (5.1%)	1 (3.7%)	
	Complex + amps	1 (4.5%)	0 (0.0%)	78 (40.0%)	9 (33.3%)	
	Simple	20 (90.9%)	32 (82.1%)	44 (22.6%)	8 (29.6%)	
	Simple + amp	1 (4.5%)	1 (2.6%)	18 (9.2%)	2 (7.4%)	
**Number of**	Multiple	1 (4.5%)	2 (5.1%)	70 (35.9%)	9 (33.3%)	**<0.001^**
**chromosomes with**	None	20 (90.9%)	32 (82.1%)	74 (37.9%)	12 (44.4%)	
**amplification**	Single	1 (4.5%)	5 (12.8%)	51 (26.2%)	6 (22.2%)	

*Fisher's exact test performed with Monte Carlo estimation

^Chi-squared test performed

Includes 8 MOC, 2 EOM, 12 MBT and 17 BEN screened by Sanger sequencing only for KRAS, BRAF and TP53, and with copy number data from SNP arrays.

Supplementary Table 6 Correlation with grade

		BEN	MBT	1	2	3	EOM	p value (FET*)
**KRAS**	MUT	12 (54.5%)	28 (71.8%)	60 (69.0%)	51 (64.6%)	12 (50.0%)	11 (40.7%)	0.066
	WT	10 (45.5%)	11 (28.2%)	27 (31.0%)	28 (35.4%)	12 (50.0%)	16 (59.3%)	
**BRAF**	MUT	0 (0.0%)	4 (10.3%)	7 (8.2%)	9 (11.5%)	1 (4.2%)	2 (7.7%)	0.66
	WT	22 (100.0%)	35 (89.7%)	78 (91.8%)	69 (88.5%)	23 (95.8%)	24 (92.3%)	
**TP53**	MUT	2 (9.1%)	7 (17.9%)	54 (62.1%)	49 (62.0%)	18 (75.0%)	11 (40.7%)	**<0.001**
	WT	20 (90.9%)	32 (82.1%)	33 (37.9%)	30 (38.0%)	6 (25.0%)	16 (59.3%)	
**CDKN2A inactivating**	Yes	5 (29.4%)	20 (51.3%)	47 (54.0%)	40 (50.6%)	14 (58.3%)	7 (25.9%)	0.061
	No	12 (70.6%)	19 (48.7%)	40 (46.0%)	39 (49.4%)	10 (41.7%)	20 (74.1%)	
**CDKN2A any (CN or**	Yes	12 (54.5%)	25 (64.1%)	70 (80.5%)	60 (75.9%)	18 (75.0%)	7 (25.9%)	**<0.001**
**mutation)**	No	10 (45.5%)	14 (35.9%)	17 (19.5%)	19 (24.1%)	6 (25.0%)	20 (74.1%)	
**CDKN2A Loss/LOH**	Loss	12 (54.5%)	22 (56.4%)	62 (71.3%)	55 (69.6%)	16 (66.7%)	5 (18.5%)	**<0.001**
	No Loss	10 (45.5%)	17 (43.6%)	25 (28.7%)	24 (30.4%)	8 (33.3%)	22 (81.5%)	
**ERBB2**	Amplified	0 (0.0%)	4 (3.7%)	24 (27.6%)	18 (22.0%)	8 (32.0%)	2 (6.5%)	**<0.001**
	Not amplified	22 (100.0%)	104 (96.3%)	63 (72.4%)	64 (78.0%)	17 (68.0%)	29 (93.5%)	
**Copy number type**	Complex	0 (0.0%)	5 (12.8%)	19 (21.8%)	17 (21.5%)	7 (29.2%)	7 (25.9%)	**<0.001^**
	Complex (whole chr)	0 (0.0%)	1 (2.6%)	3 (3.4%)	5 (6.3%)	2 (8.3%)	1 (3.7%)	
	Complex + amps	1 (4.5%)	0 (0.0%)	25 (28.7%)	39 (49.4%)	13 (54.2%)	9 (33.3%)	
	Simple	20 (90.9%)	32 (82.1%)	29 (33.3%)	12 (15.2%)	1 (4.2%)	8 (29.6%)	
	Simple + amp	1 (4.5%)	1 (2.6%)	11 (12.6%)	6 (7.6%)	1 (4.2%)	2 (7.4%)	
**Number of**	Multiple	1 (4.5%)	2 (5.1%)	22 (25.3%)	34 (43.0%)	13 (54.2%)	9 (33.3%)	**<0.001^**
**chromosomes with**	None	20 (90.9%)	32 (82.1%)	41 (47.1%)	24 (30.4%)	7 (29.2%)	12 (44.4%)	
**amplification**	Single	1 (4.5%)	5 (12.8%)	24 (27.6%)	21 (26.6%)	4 (16.7%)	6 (22.2%)	

*Fisher's exact test performed with Monte Carlo estimation

^Chi-squared test performed

Includes 8 MOC, 2 EOM, 12 MBT and 17 BEN screened by Sanger sequencing only for *KRAS*, *BRAF* and *TP53*, and with copy number data from SNP arrays.

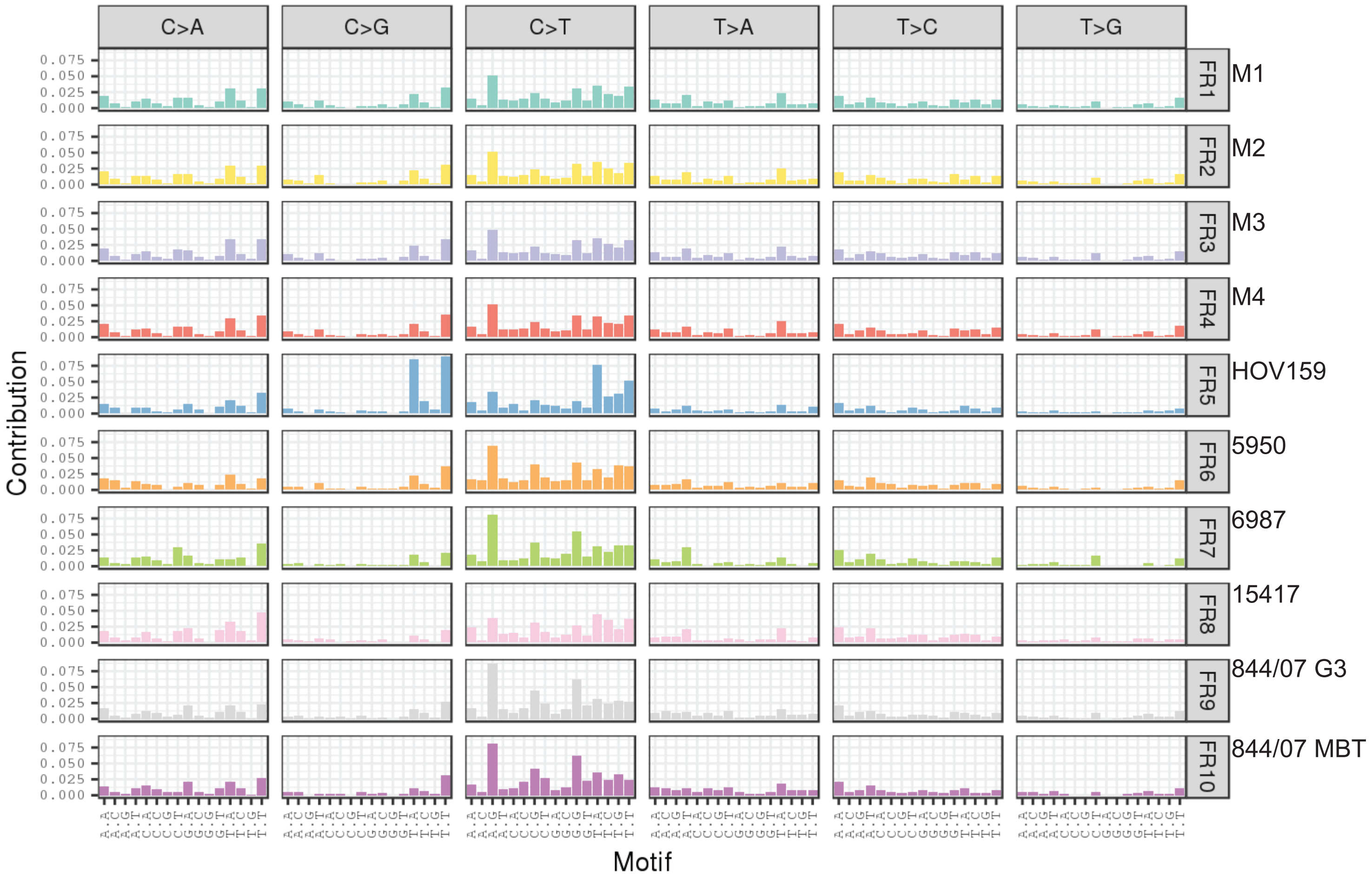

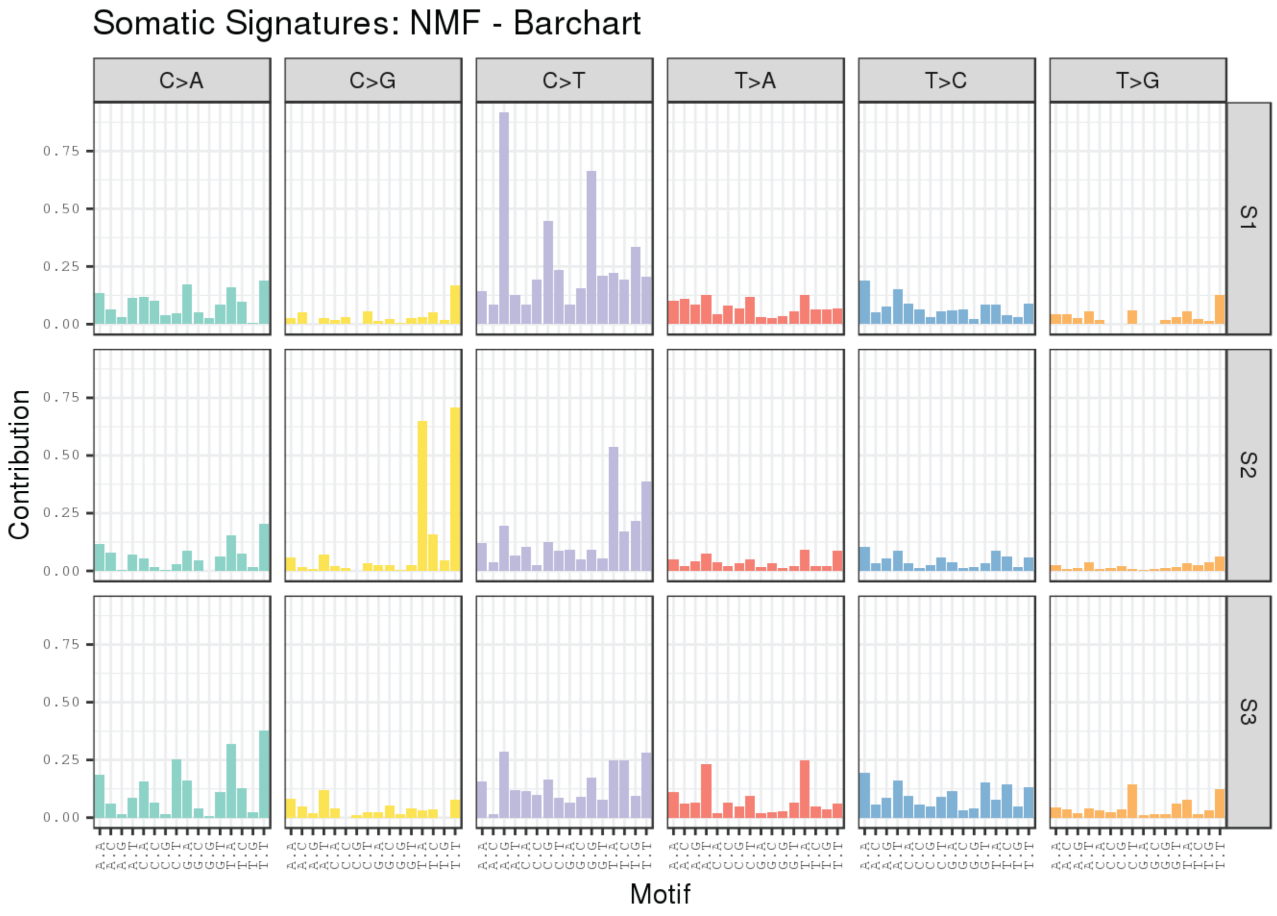

Supplementary Figure 6. De novo mutation signature detection of whole genome sequencing samples. Top: Variant trinucleotide profiles of cases. M1 - M4, 4 metastatic sites from rapid autopsy case. Bottom: Signatures predicted.

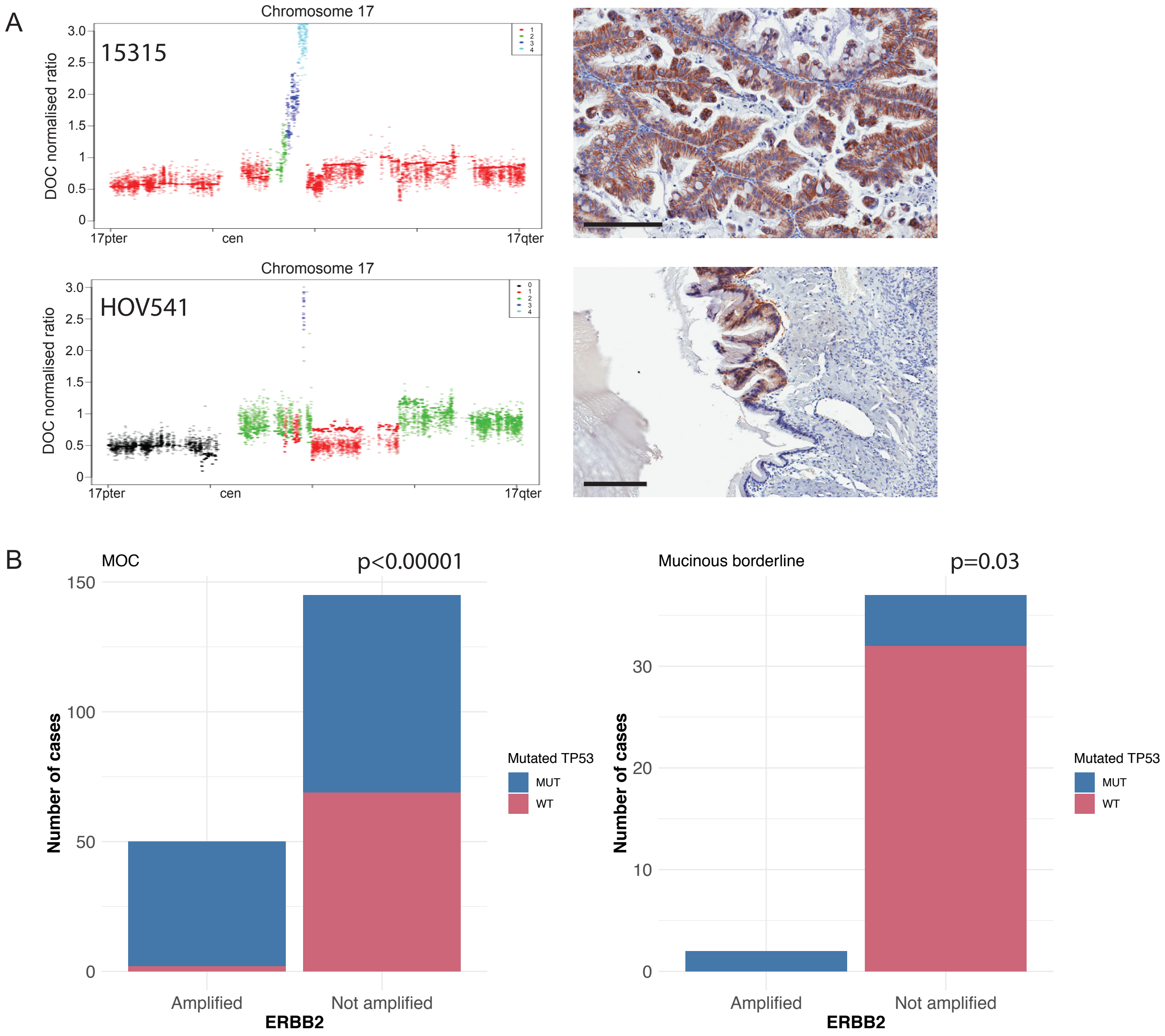

Supplementary Figure 7. ***ERBB2*** **amplification**
A. Concordance of IHC (images at right) with copy number by array/sequencing (chromosome 17 profiles at left). 15315 - case with perfect concordance of 3+ staining and high level amplification. HOV541 - case with concordance of 3+ staining and high level amplification, but showing negative staining in nearby area of non-malignant epithelium. Scale bars 200 μm.
B. Correlation of *ERBB2* amplification (IHC/CISH or copy number) with mutated *TP53*. *ERBB2* amplified MOC are strongly associated with mutant *TP53*, whereas this is a rarer combination in mucinous borderline tumours. P values from two-sided Fisher's exact test: MOC OR 21.54, 95% CI 5.3-189.3; MBL OR Inf, 95% CI 0.93-Inf. Difference in the combination of *TP53* mutated/*ERBB2* amplified versus not, comparing MOC with borderline, 0=0.0049, two-sided Fisher's exact test, OR 0.17, 95%CI 0.02-0.69.

A

Ensembl.ID	Chromosome	Gene.start.bp	Gene.end.bp	Gene.name	Amplicon	No_Amplicon	FDR	AveExpr	P value
ENSG00000107341	9	33817567	33920404	UBE2R2	0	-1.36813	0.00002	6.10965	1.53E-09
ENSG00000137073	9	33921693	34048949	UBAP2	0	-1.65231	0.00004	6.31700	4.93E-09
ENSG00000198876	9	34086387	34127399	DCAF12	0	-1.44861	0.00004	6.08446	8.10E-09
ENSG00000164978	9	34329506	34343713	NUDT2	0	-1.93047	0.00025	3.62134	6.78E-08
ENSG00000137100	9	34613545	34620523	DCTN3	0	-1.10607	0.00026	4.03182	8.79E-08
ENSG00000186638	9	34252381	34311371	KIF24	0	-2.38225	0.00041	3.09456	1.65E-07
ENSG00000164967	9	34610486	34612104	RPP25L	0	-1.58068	0.00199	3.38779	9.38E-07
ENSG00000147955	9	34634722	34637809	SIGMAR1	0	-1.45988	0.01645	4.80132	9.63E-06
ENSG00000186871	X	72204657	72239047	ERCC6L	0	-2.29417	0.01645	2.25817	9.98E-06
ENSG00000165006	9	34179005	34252523	UBAP1	0	-1.00380	0.02245	5.76266	1.51E-05
ENSG00000123975	9	89311198	89316703	CKS2	0	-2.02474	0.02583	3.79337	1.92E-05
ENSG00000178691	17	31937018	32001045	SUZ12	0	-1.25460	0.03973	6.14738	3.58E-05
ENSG00000164109	4	120055608	120067074	MAD2L1	0	-1.93745	0.03973	3.65113	3.60E-05
ENSG00000123219	5	65517766	65563171	CENPK	0	-1.91351	0.03973	3.30296	3.83E-05
ENSG00000111247	12	4538798	4560048	RAD51AP1	0	-1.94434	0.03973	3.04424	4.13E-05
ENSG00000108651	17	31860899	31901765	UTP6	0	-0.99733	0.03973	5.68644	4.29E-05
ENSG00000126858	17	32142454	32253374	RHOT1	0	-0.99427	0.04623	5.78200	5.30E-05
ENSG00000176208	17	30831970	30895869	ATAD5	0	-1.89710	0.04739	4.32448	5.83E-05
ENSG00000198056	12	56731596	56752373	PRIM1	0	-1.34491	0.04739	3.34625	6.37E-05
ENSG00000134291	12	47963569	47968878	TMEM106C	0	-1.80443	0.04739	6.02772	6.39E-05
ENSG00000185480	12	102120185	102197520	PARPBP	0	-1.71474	0.04882	3.51519	6.91E-05

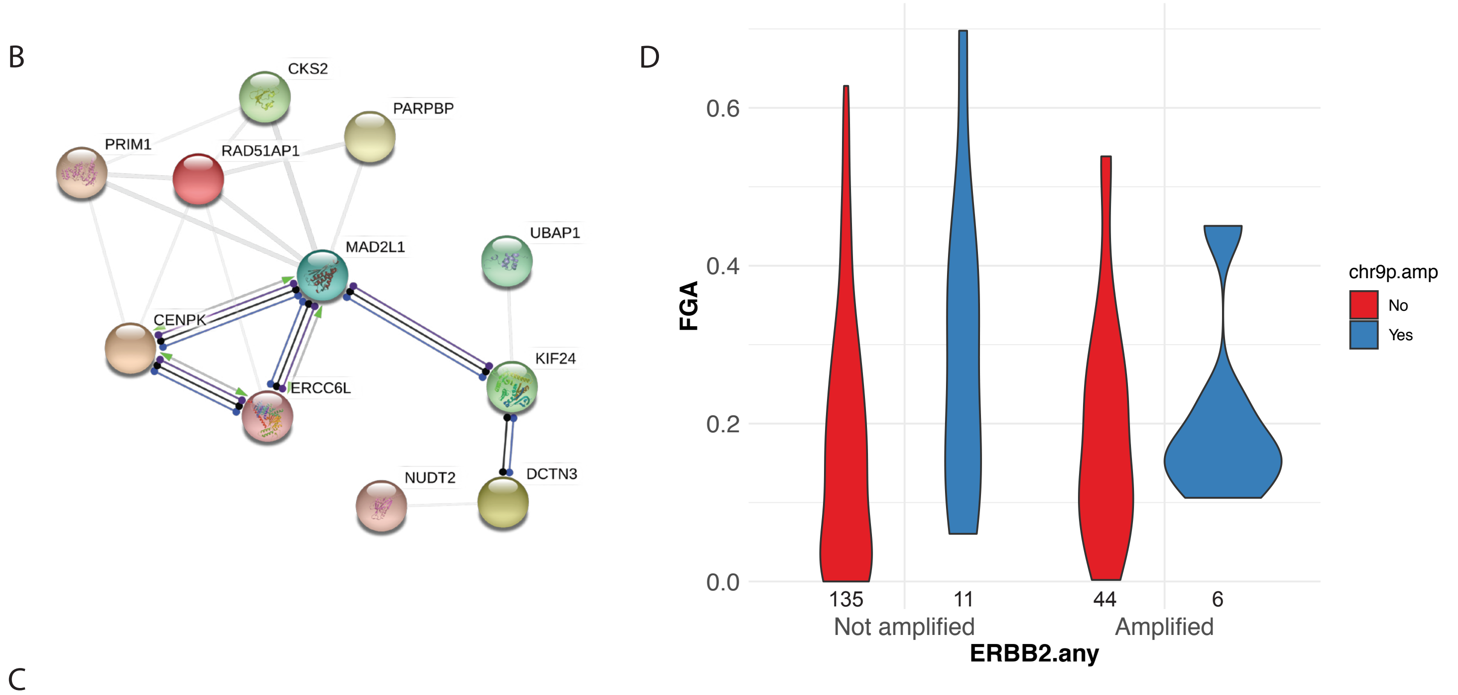

C

#pathway ID	pathway description	observed gene count	false discovery rate	matching proteins in your network (labels)
GO.0000777	condensed chromosome kinetochore	4	0.0015	CENPK,DCTN3,ERCC6L,MAD2L1
GO.0000779	condensed chromosome, centromeric region	4	0.0015	CENPK,DCTN3,ERCC6L,MAD2L1
GO.0005694	chromosome	7	0.0015	CENPK,DCTN3,ERCC6L,MAD2L1,PARPBP,PRIM1,SUZ12
GO.0044427	chromosomal part	7	0.0015	CENPK,DCTN3,ERCC6L,MAD2L1,PARPBP,PRIM1,SUZ12
GO.0000793	condensed chromosome	4	0.00618	CENPK,DCTN3,ERCC6L,MAD2L1

Supplementary Figure 8. 9p Amplicon RNAseq analysis

A: Gene differentially expressed by Degust between 9p amplicon containing and absent cases.

B: STRING network (unconnected nodes not shown).

C: GO terms enriched in network (determined by STRING).

D: Comparison of fraction genome altered by copy number (FGA) by amplification status. The presence of the 9p ampliconis associated with a statistically significant increase in FGA (ANOVA + Tukey post test p=0.037 F value = 4.435, diff 0.08, 95% CI 0.005-0.16), which was not the case for *ERBB2* amplification (p=0.66 F value = 0.19, diff -0.01, 95% CI 0.06-0.039). Number of MOC in each group indicated below. A model that included grade still showed a significant association for 9p amplification and FGA (ANOVA + Tukey post test, p=0.02, diff 0.087, 95% CI 0.014-0.16).

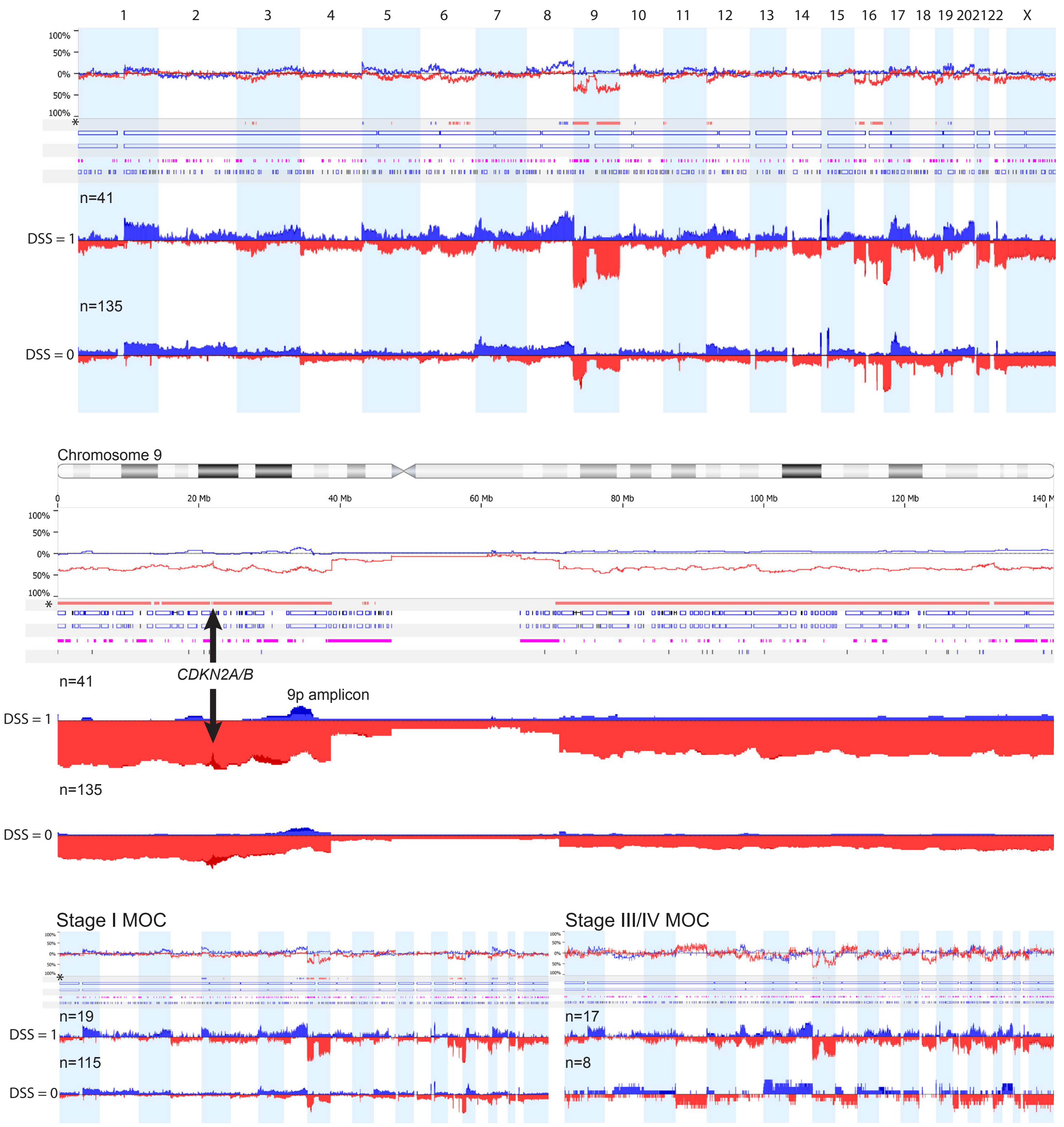

Supplementary Figure 9. Differences in copy number based on disease specific survival status (DSS, 1 = deceased, 0 = alive). Gains in blue, losses in red. In each panel, the difference plot it above and the individual frequency plots for each group below. *Red bars indicate significantly different losses and blue bars indicate significantly different gains at $p<0.05$. Top - all MOC genome-wide. Middle - all MOC, chromosome 9 only. Bottom, MOC subdivided by stage, genome-wide.

c. Mutational signatures in MOC

Mutation signatures were only derived for paired exome samples (n=41). As stated in the main results, COSMIC signature 1 was the most common dominant signature in MOC. For many cases (18/41) there was no single signature assigned that encompassed >40% of variants. Mismatch repair (MMR)-related signatures (Sigs 6, 15 and 26) were frequently observed (71% of MOC, 21/41) most often as minor components (10-40% of variants). We do not think the presence of these signatures indicates a true mismatch repair deficiency due to the lack of any detectable germline or somatic MMR gene mutations or hypermutation in these cases. Their presence is perhaps indicative of a similarity in mutational processes to colorectal, gastric or endometrial tumors that would have been represented in the building of this mutation signature. The exception was a single MOC in the validation cohort that carried an *MSH3* mutation and high mutation load (0.5% of MOC overall).

d. Structural analysis

As described, we identified five general copy number profile types: simple (≤4 chromosomes affected, 23% of cases), simple with one high-level amplification (9% of cases, 11/17 of which were *ERBB2* amplified), complex (23% of cases, > 4 chromosomes affected), complex-whole chromosome (5% of cases, >4 chromosomes but with few intra-chromosomal breakpoints) and complex with multiple high-level amplifications (40% of cases).
The most common amplicon, targeting *ERBB2*, was most often associated with one of the complex profiles (78% of amplified cases), similar to observations in gastric cancer but different from breast cancer (1, 2).

Measures of genomic instability were correlated with mutational data. The copy number type, number of chromosomes with high-level copy number aberrations and fraction of the genome

altered (FGA) by copy number were not positively associated with mutated *KRAS* either as categorical or numerical features in MOC. *TP53* mutation was strongly associated with these features in MOC.

Supplementary Table 7: Association of genomic instability measures with mutation status

Feature	*KRAS* mut/wt	P value	*TP53* mut/wt	P value	*ERBB2* amp/not	P value
Mean FGA	16%/23%	0.045[1]	22%/12%	<0.001[1]	19.4%/18.3%	0.34[1]
Mean # chr with amplifications	1.17/2.14	0.265[1]	2.08/0.55	<0.001[1]	2.47/1.19	<0.001[1]
FGA.cat (<10% >10%)		0.46[2]		<0.001[2]		<0.001[2]
# chr with amplifications (None, one, multiple)		0.58[2]		<0.001[2]		<0.001[2]
CN type		0.76[3]		<0.001[3]		<0.001[3]

1. (Wilcoxon rank sum test) 2. Fisher's exact test 3. Chi-squared test. Source data in Supplementary Table 1.

The 9p13.3 amplicon was present in two high-grade carcinomas (HOV159 and 5950) for which we had WGS data. The mechanism of amplification differed between these cases, with the structural rearrangement data for HOV159 mostly internal to chromosome 9, comprising tandem duplication and a complex series of deletions (encompassing *CDKN2A*) and inversions (Fig 3). In contrast, 5950 had fold-back inversions and more external breakpoint partners (Fig 3).

Overall, the WGS data is summarised below. None had a profile of high numbers of small tandem duplications suggesting homologous recombination deficiency (3). Patient 5950 had a profile with many small (<1 Mb) inversions, deletions and duplications (64.5% of SV) suggesting mostly intra-chromosomal breakage-fusion-bridge cycles, whereas HOV159 and 15407 tended to have larger intra-chromosomal events (34% and 35% of structural variants), and 6987 had high levels of inter-chromosomal translocations (38.5%). The primary tumor for the rapid autopsy case (844/07) had fewer structural variations, and similar to 5950 these were most often small intra-chromosomal events, although with fewer small duplications

than 5950. The metastatic sites of this case (M1-4) were similar to each other, and had many more structural variants than the primary tumors. In particular they had an increase in foldback inversions consistent with the presence of multiple high-level amplifications.

Supplementary Table 8: Structural variant categories based on Shah *et al.* (3)

Sample	Del <1Mb	Dup <1Mb	INV (30-1Mb)	Unbalanced (DUP, DEL, INV >1 Mb)	FB INV (<30 kb)	Balanced Translocation	Total number
6987	17 (9.5%)	7 (3.9%)	22 (12.3%)	114 (63.7%)	18 (10.1%)	1 (0.6%)	179
5950	31 (29.0%)	11 (10.3%)	12 (11.2%)	38 (35.5%)	15 (14.0%)	0 (0.0%)	107
HOV159	52 (26.5%)	5 (2.6%)	12 (6.1%)	108 (55.1%)	14 (7.1%)	5 (2.6%)	196
15407	27 (17.2%)	9 (5.7%)	14 (8.9%)	89 (56.7%)	16 (10.2%)	2 (1.3%)	157
844/07 MBT	26 (38.2%)	2 (2.9%)	9 (13.2%)	25 (36.8%)	6 (8.8%)	0 (0.0%)	68
844/07 G3	17 (28.8%)	1 (1.7%)	9 (15.3%)	28 (47.5%)	4 (6.8%)	0 (0.0%)	59
M1	38 (25.5%)	5 (3.4%)	11 (7.4%)	73 (49.0%)	20 (13.4%)	2 (1.3%)	149
M2	35 (25.7%)	6 (4.4%)	11 (8.1%)	68 (50.0%)	16 (11.8%)	0 (0.0%)	136
M3	33 (23.9%)	4 (2.9%)	12 (8.7%)	70 (50.7%)	18 (13.0%)	1 (0.7%)	138
M4	40 (27.4%)	6 (4.1%)	12 (8.2%)	69 (47.3%)	17 (11.6%)	2 (1.4%)	146

Supplementary Table 9: Alternative summary of structural variants

Sample	Small intra-choromosomal (<1Mb)	Large intra-chromosomal (>1 Mb)	Inter-chromosomal	Total number
6987	64 (35.8%)	46 (25.7%)	69 (38.5%)	179
5950	69 (64.5%)	18 (16.8%)	20 (18.7%)	107
HOV159	83 (42.3%)	67 (34.2%)	46 (23.5%)	196
15407	66 (42.0%)	55 (35.0%)	36 (22.9%)	157
844/07 MBT	43 (63.2%)	8 (11.8%)	17 (25.0%)	68
844/07 G3	31 (52.5%)	10 (16.9%)	18 (30.5%)	59
M1	74 (49.7%)	36 (24.2%)	39 (26.2%)	149
M2	68 (50.0%)	35 (25.7%)	33 (24.3%)	136
M3	67 (48.6%)	33 (23.9%)	38 (27.5%)	138
M4	75 (51.4%)	35 (24.0%)	36 (24.7%)	146

e. Rapid autopsy case

The patient was aged 41 years at diagnosis, and initially presented with elevated CA125 and CA19-9 and a large mass on imaging. She underwent surgery to remove the 100 mm diameter tumor, which was localized to the ovary (Stage IA) and appeared to have an intact capsule. Some of the omentum, the opposite ovary, both fallopian tubes, uterus and cervix

were also removed, with no abnormalities detected. The initial pathology report stated that most of the tumor was borderline, with small areas of intraepithelial carcinoma and invasive carcinoma, mostly expansile invasion, but small areas of infiltrative invasion were noted. Post-surgical imaging (PET/CT) did not identify any overt metastatic disease. No chemotherapy was administered.

After 21 months, her serum markers were still normal, however at 26 months after surgery, she presented with abdominal discomfort and elevated CA125 and CA19-9 (Figure 4). A CT scan indicated omental thickening, ascites and pleural effusion. Her rapidly deteriorating condition meant that no chemotherapy was given, and death followed within a month of the recurrence. She had significant co-morbidities, including obesity and Type II diabetes (diagnosed two years before the cancer); the latter was not controlled at recurrence. On autopsy, disease was widespread with peritoneal, pleural, rectus abdominis muscle and lymph node metastases. Sixteen tissue specimens were collected at autopsy (Figure 4). The pancreas was sampled without finding any abnormalities.

Pathology review of the initial tumor confirmed that this was likely an ovarian primary, further supported by immunohistochemistry, which showed CK7+, CK20+, CDX2 focally positive, PAX8 and ER negative. Histopathological analysis of the primary and recurrence tumors identified a small area in the primary tumor that had high grade characteristics, which matched the histology of the recurrence sites. All recurrence sites showed high grade, infiltrative tumors (Figure 4).

DNA and RNA was extracted from dissected frozen tissue, including separate areas of borderline and high-grade morphology in the primary tumor. Whole genome sequencing was

performed on the two primary areas and four metastatic sites: omentum (M1), right iliac lymph node (M3), diaphragm (pleural side, M2) and para-aortic lymph node (M4).

De novo mutational signature detection found that the primary tumor samples were very similar, mostly comprised of signature S1 (related to COSMIC Sig 1). However, the four samples from metastases had quite a different composite signature, with a reduced contribution from S1, and an increased contribution from S3 and to a lesser extent S2 (APOBEC, COSMIC Sigs 2 and 13). The metastatic sites were very similar to each other, as reflected in the Sanger sequencing data across all 15 available sites shown in the table below. The variation in allele frequencies observed across these samples can be explained by differing levels of normal cell contamination in each.

Supplementary Table 10: Sanger sequencing validation of variants in primary borderline (BDL) sample and 15 metastatic sites. Highlighted in blue are the sites that underwent WGS. Colours indicate the percentage of the allele in the sample, estimated from the electrophoretogram.

Site:	BDL primary	R pleura	pleural diaphagm	LN L lung	L pleura	rectus muscle	L para-aortic LN	R iliac LN	small bowel	R iliac LN (B)	R para-aortic LN	Omental	Omental	porta-hepatic LN	peritoneal surface	anterior mediastinal
chr11:54014453	0	0	1	0	0	0	0	0	0	0	DNW	0	0	0	0	0
HS6ST3	0	0	1	1	0	0	1	1	2	1	1	1	1	1	1	1
chr1:101400874	0	0	1	1	0	1	1	1	1	1	1	1	1	1	1	1
chr8:114907557	0	0	1	0	0	1	1	1	1	1	1	2	1	2	1	1
NFIB	0	0	1	1	1	1	1	1	1	1	1	2	1	1	1	1
TRAF3IP3	0	0	0	1	1	1	1	1	1	1	1	1	1	1	1	1
RP11-473J6.1	0	1	1	2	1	1	1	1	1	1	1	1	1	1	1	1
RP11-401O9.3	0	1	2	2	2	1	1	2	2	2	2	2	2	2	2	2
PCDH11X	0	1	3	1	2	3	2	4	4	4	4	3	2	2	2	2
PIK3R6	0	1	4	3	2	2	2	3	2	3	2	2	2	2	2	2
FAM35BP	1	1	1	1	1	1	1	1	1	1	1	2	2	1	1	1
MIR320A	3	3	3	3	3	3	3	4	3	3	3	3	3	3	3	3
TP53	2	2	3	2	3	2	3	3	4	4	4	4	4	4	4	4

0	<5%
1	5-25%
2	25-50%
3	50-75%
4	>75%

Supplementary Table 11. Primer sequences

Gene	Left Primer	Right Primer	Product size
ARID1A_Exon 16	TGAGGGAAACATGAGCACTG	GGAGGCAAAGAAACACAGGA	212
ARID1A_Exon 20	TTACACTCGCCAACATCTCG	CACATTGTTGTCCTGGATGC	196
BRAF_V600E	CCTAAACTCTTCATAATGCTTGCTC	CCACAAAATGGATCCAGACA	189
CDKN2A_Exon 1	AGCACCGGAGGAAGAAAGAG	AGCGCTACCTGATTCCAATTC	351
CDKN2A_Exon 2	GTGAGGGGGCTCTACACAAG	ACCAGCGTGTCCAGGAAG	213
CTCFMRE	AGCTCGATTTCTCCTGCAGA	AAGCCTAAGTCCCACTGCC	197
EFHC2	CATGGAGGGAGTTTGTTGCC	CCCCTTCTCTTGTATCATTGCTG	294
ERBB2_Exon19	CCCACGCTCTTCTCACTCAT	AGAGACCAGAGCCCAGACCT	212
ERBB2_Exon20	GCTGTGGTTTGTGATGGTTG	TCCCGGACATGGTCTAAGAG	213
FAM35BP	ATCCTCTCATGTCAGCCTCC	CACGGGTGAGGTCATTATGG	449
HS6ST3	GCTGGTGACCTTGCCTCTTA	GGAGGAAGAGGAAGGGCAAT	193
Intergenic variant chr1:101400874	GGCTCCTGGACTTGAACTCT	GACCATTGTGAGGAAGGTCG	162
Intergenic variant chr11:54014453	GAGGCCTTCGGTGGAAAAG	GCTTCCGTGTAGTTCTGGGA	390
Intergenic variant chr8:114907557	TGTCTTTCATGGCACTGGTAT	GGGAATGCAGATCAGTTGATGG	275
KRAS_codons12/13	AGGCCTGCTGAAAATGACTG	CATGAAAATGGTCAGAGAAACC	205
LOC105372877	AGCCACTCTTTCCATCCTCC	AATAGTAGCCGGGCCTGG	533
LOC105377582	TAAGTTTCCTGTGGCCTCCC	TTCCCGCATCTTCCTGTCTT	247
MIR320A	TGGGCTCAATCGATCCTCTC	TGAGATTGCGCTGGAGTGTA	246
NFIB	CAGCCTGAGAGAAATGCTGC	TGAGAGGAGGAAGTAGCAAGG	202
NINL	TAAAGGGACAGGAAGGGCAG	CCTAGGTCGAAGCAGTCACA	178
PCDH11X	CAGGCAAAGGTAGGCAGGAA	GAGAGAAGCTATCCAAGGCAGT	223
PIK3CA_Exon 20	CTCTGGAATGCCAGAACTAC	ATGCTGTTTAATTGTGTGGAAG	175
PIK3CA_Exon 9	CAGAGTAACAGACTAGCTAGAGAC	CCACAAATATCAATTTACAACCATTG	309
PIK3R6	TCACCCAAAACCTTGCAAGC	GCTTTGCAGTCTTGTGTAATTGT	296
RNF43_Exon 10	CCTCTCAGCCACACTCTGC	GACTCTGTGCCAGGTAGGG	201
RNF43_Exon 2	CCTGCATTTGGTATTAGCTAGTGG	GCAGTAGAAGCCCGTGTATGG	416
RNF43_Exon 3	CTCACAGCTCACTCCTGCTG	ACATGGGGACAAGAGAGCAC	268
RNF43_Exon 4	ATGTGTGAGGGGTGAGGCTA	GTGACTTCTCCCTGCCCTTC	246
RNF43_Exon 5	CTCAGGGGAGAGGGAAGG	TGCCACAGGACAAAGTAGGG	254
RNF43_Exon 6	CGAATTGACCCAGCTCATCC	TCCTTCCTTCTCCCTAACCAC	237
RNF43_Exon 7	CAAGCTTGGGTCCAGAGAGG	CTCCCCCAGCTTCAATCTCC	291
RNF43_Exon 8	CCTGGCAATTCCTATGGCTA	CCAAAGGGAAGCCACATTC	226
RNF43_Exon 9A	GAGCTCACAGGCTACTCAGG	ATCTGCCAGGTACCCACTGC	540
RNF43_Exon 9B	CAGCACCCCTATGCACAAGG	GAGGAATAGGAGGCCTGGAC	526
RNF43_Exon 9C	TCCTTGGACTCGGTGGTG	GGACCAAGGATATGCCACAC	486
RNF43_Exon 9D	CAAAAATCCAGCCTCTCTGC	GCTGTAGTCTCCTCTCCCTA	430
RP11-401O9.3 (myosin-13)	CTGCCCGTTTCTGAGATGTG	TGACTCCTCACTAACCAGCG	176
RP11-473J6.1	AGCCTGGAATCTCTCTTGCT	TTTGCCTCACAGTTCTGCAG	162
SRGAP2B	TCTCACTCTATCACCAGGCTG	AGGAGTTCGAGATCATCCCG	202
TP53_Exon 4	CCTGGTCCTCTGACTGCTCTTTTCACCCA	GGCCAGGCATTGAAGTCTCAT	363
TP53_Exon 5	CACTTGTGCCCTGACTTTCA	AACCAGCCCTGTCGTCTCT	267
TP53_Exon 6	AGAGACGACAGGGCTGGTTG	CTTAACCCCTCCTCCCAGAG	216
TP53_Exon 7	CCTGCTTGCCACAGGTCT	GTGTGCAGGGTGGCAAGT	201
TP53_Exon 8	TTTCCTTACTGCCTCTTGCTTC	TAACTGCACCCTTGGTCTCC	227
TP53_Exon 9	GGAGACCAAGGGTGCAGTTATGCCTCAG	CCCAATTGCAGGTAAAACAG	231
TRAF3IP3	TGACTCCCACACCAATGACA	TGGGGTCTCACTTTGTCACC	325

Supplementary References

1. Liu Y, Sethi NS, Hinoue T, Schneider BG, Cherniack AD, Sanchez-Vega F, et al. Comparative Molecular Analysis of Gastrointestinal Adenocarcinomas. Cancer Cell. 2018;33(4):721-35 e8.
2. Staaf J, Jonsson G, Ringner M, Vallon-Christersson J, Grabau D, Arason A, et al. High-resolution genomic and expression analyses of copy number alterations in HER2-amplified breast cancer. Breast Cancer Res. 2010;12(3):R25.
3. Wang YK, Bashashati A, Anglesio MS, Cochrane DR, Grewal DS, Ha G, et al. Genomic consequences of aberrant DNA repair mechanisms stratify ovarian cancer histotypes. Nature genetics. 2017;49(6):856-65.
