# Supplementary material for: The molecular origin and taxonomy of mucinous ovarian carcinoma
Source: Nat Commun. 2019 Sep 2;10:3935. doi: 10.1038/s41467-019-11862-x (PMC6718426; doi:10.1038/s41467-019-11862-x)
Supplement: Supplementary file 3 — Description of Additional Supplementary Files [file 41467_2019_11862_MOESM3_ESM.pdf]

Description of Additional Supplementary Files

File Name: Supplementary Data 1
Description: Cohort information

File Name: Supplementary Data 2
Description: Genes targeted by validation sequencing panel

File Name: Supplementary Data 3
Description: Regions of copy number differentially affected by grade, stage, outcome and classification, plus source data for copy number variants

File Name: Supplementary Data 4
Description: Sequencing quality control metrics

File Name: Supplementary Data 5
Description: Variants from exome and validation sequencing

File Name: Supplementary Data 6
Description: MANTA output from WGS
