# Supplementary material for: The molecular origin and taxonomy of mucinous ovarian carcinoma
Source: Nat Commun. 2019 Sep 2;10:3935. doi: 10.1038/s41467-019-11862-x (PMC6718426; doi:10.1038/s41467-019-11862-x)
Supplement: Supplementary file 10 — Reporting Summary [file 41467_2019_11862_MOESM10_ESM.pdf]

nature research

Corresponding author(s): Kylie Gorringe

Reporting Summary

Nature Research wishes to improve the reproducibility of the work that we publish. This form provides structure for consistency and transparency in reporting. For further information on Nature Research policies, see Authors & Referees and the Editorial Policy Checklist.

Statistical parameters

When statistical analyses are reported, confirm that the following items are present in the relevant location (e.g. figure legend, table legend, main text, or Methods section).

n/a	Confirmed	
☐	☒	The exact sample size (*n*) for each experimental group/condition, given as a discrete number and unit of measurement
☐	☒	An indication of whether measurements were taken from distinct samples or whether the same sample was measured repeatedly
☐	☒	The statistical test(s) used AND whether they are one- or two-sided<br>*Only common tests should be described solely by name; describe more complex techniques in the Methods section.*
☐	☒	A description of all covariates tested
☐	☒	A description of any assumptions or corrections, such as tests of normality and adjustment for multiple comparisons
☐	☒	A full description of the statistics including central tendency (e.g. means) or other basic estimates (e.g. regression coefficient) AND variation (e.g. standard deviation) or associated estimates of uncertainty (e.g. confidence intervals)
☐	☒	For null hypothesis testing, the test statistic (e.g. *F*, *t*, *r*) with confidence intervals, effect sizes, degrees of freedom and *P* value noted<br>*Give P values as exact values whenever suitable.*
☒	☐	For Bayesian analysis, information on the choice of priors and Markov chain Monte Carlo settings
☒	☐	For hierarchical and complex designs, identification of the appropriate level for tests and full reporting of outcomes
☒	☐	Estimates of effect sizes (e.g. Cohen's *d*, Pearson's *r*), indicating how they were calculated
☐	☒	Clearly defined error bars<br>*State explicitly what error bars represent (e.g. SD, SE, CI)*

Our web collection on statistics for biologists may be useful.

Software and code

Policy information about availability of computer code

Data collection	No software was used to collect the data
Data analysis	Data was analysed using Nexus Copy Number (BioDiscovery Inc. v8), R 3.3.0, STRING v10.5, Geneious 8.1.9 (Biomatters, Auckland, New Zealand), Primer 3, ADTEx (v1), MANTA (v1.0.3), FACETs(v0.5.6), CopywriteR (v1), DeconstructSigs (v 1.8.0), SomaticSignatures (v1), VarDict (v1.4.6), MuTect2 (v3.5), GATK HaplotypeCaller (v3.2), BWA-MEM (v0.7.10), Picard (v1.119), gplot v 3.0.1, Degust (v3)

For manuscripts utilizing custom algorithms or software that are central to the research but not yet described in published literature, software must be made available to editors/reviewers upon request. We strongly encourage code deposition in a community repository (e.g. GitHub). See the Nature Research guidelines for submitting code & software for further information.

Data

Policy information about availability of data

All manuscripts must include a data availability statement. This statement should provide the following information, where applicable:

- Accession codes, unique identifiers, or web links for publicly available datasets
- A list of figures that have associated raw data
- A description of any restrictions on data availability

All sequencing data is available from the European Genome-phenome Archive (https://ega-archive.org/studies) with EGA Study ID EGAS00001003545

Field-specific reporting

Please select the best fit for your research. If you are not sure, read the appropriate sections before making your selection.

☒ Life sciences ☐ Behavioural & social sciences ☐ Ecological, evolutionary & environmental sciences

For a reference copy of the document with all sections, see nature.com/authors/policies/ReportingSummary-flat.pdf

Life sciences study design

All studies must disclose on these points even when the disclosure is negative.

Sample size	Sample size was determined by availability of sample tissue.
Data exclusions	Data were excluded if sequencing metrics failed quality control.
Replication	Validation of sequencing findings was performed by an alternative method (Sanger sequencing) for key genes mentioned in the study. A proportion of cases with ERBB2 amplification were verified by CISH. Other findings were unable to be replicated due to the lack of a validation cohort (e.g. survival analyses).
Randomization	Not relevant to the study, cases are grouped for analysis by clinico-pathological features
Blinding	Not relevant to the study, cases are grouped for analysis by clinico-pathological features

Reporting for specific materials, systems and methods

Materials & experimental systems

n/a	Involved in the study	
☒	☐	Unique biological materials
☒	☐	Antibodies
☒	☐	Eukaryotic cell lines
☒	☐	Palaeontology
☒	☐	Animals and other organisms
☐	☒	Human research participants

Methods

n/a	Involved in the study	
☒	☐	ChIP-seq
☒	☐	Flow cytometry
☒	☐	MRI-based neuroimaging

Human research participants

Policy information about studies involving human research participants

Population characteristics	Participants were female, of any age, with a diagnosis of a mucinous ovarian tumour between 1987 and 2015.
Recruitment	Participants were recruited from hospitals in Australia, UK, USA and Canada. There are unlikely to be any biases relevant to the study as they were recruited at first diagnosis and are representative of the ovarian cancer population.
